# Supplementary material for: Are cause of death data fit for purpose? evidence from 20 countries at different levels of socio-economic development
Source: PLoS One. 2020 Aug 24;15(8):e0237539. doi: 10.1371/journal.pone.0237539 (PMC7446871; doi:10.1371/journal.pone.0237539)
Supplement: S1 File — (DOCX) [file pone.0237539.s001.docx]

## S1 File

**What is ANACONDA?**

ANACONDA is a new tool for checking the quality of ICD coded mortality data and for supporting country efforts to improve COD certification practices. It provides a step-by-step approach to enable users to quickly conduct a comprehensive review of data on mortality levels and causes of death.

In particular, the tool allows users to identify problems in the data caused by incomplete reporting/registration of deaths in the population, poor certification practices, and overall poor performance of the COD reporting system. Through a series of computational steps ANACONDA helps users understand how fit–for-purpose their data are. Completeness of the dataset is estimated with a new empirical method; because the statistical model in the method it uses the logit of completeness, for some countries with a high estimated completeness (e.g. 98%) it can be assumed to be 100%. Cause of death codes in the data that are incorrectly used for certifying and coding cause of death data – the so-called “garbage codes” are identified, and an overall indicator of the quality of the output of the vital statistics reporting system (VSPI(Q)) is calculated.

The tool is designed around 10 STEPS, these in turn can be grouped under 4 broad categories:

*1. Data inputs and general background checks (Step 1)*

*2. Mortality data (Steps 2-5)*

*3. Cause of death data (Steps 6-9)*

*4. Overall data quality index: VSPI(Q) (Step 10)*

The Vital Statistics Performance Index for Quality, or VSPI(Q), is a summary score of overall system performance that considers five essential components of quality:

1. Completeness of death registration
2. Quality of cause of death reporting (fraction of unusable or insufficiently specified codes)
3. Level of cause-specific detail available (amount of detail in the cause of death list used for tabulation)
4. Quality of age and sex reporting (extent to which age and/or sex are missing in the data)
5. Number of biologically implausible underlying causes.

Scores on each of these five components are weighted according to their importance in determining the correct COD distribution in a population and combined into a total VSPI(Q) score (0–‍100 per cent). The higher the score, the better the overall quality of the mortality data. Values above 85 per cent suggest a well-functioning CRVS system that will meet most policy needs for reliable data.

The optimal source of such mortality statistics is a civil registration system covering the entire country, with all deaths being medically certified as to the underlying cause, but ANACONDA can be applied to any mortality dataset, as long as the data are by age and sex and coded to ICD-10, 3rd or 4th digit. To benefit from all the checks and computations performed in the first 5 steps, data for the corresponding population at risk, by age and sex, also need to be entered. The tabulated data and graphs are assessed for plausibility by checking them against fundamental demographic and epidemiological relationships or against comparators from respected global sources to assess whether the input data are roughly consistent with these estimates.

**Garbage codes in ANACONDA:**

**ANACONDA uses the extensive list of garbage codes defined by the Global Burden of Diseases (GBD) study but classifies these into different typologies to better support the targeting of public health strategies to reduce garbage codes and improve the evidence base about disease and injury patterns and intervention control strategies. Broadly speaking garbage codes can be divided into** two types, those that do not tell us anything about the true underlying cause and therefore can be referred to as being totally “unusable” and those which have a valid ICD code, but which are “insufficiently specified”. The latter have some usable information content and hence are less bad but by not indicating for example, the site of the cancer or what type of stroke, important information for the health care system is missed. This distinction into two types makes it possible to subsequently weight them differently when calculating summary indices of overall data quality in ANACONDA.

Two distinct typologies are used in ANACONDA to provide useful insight into error patterns that can assist countries in design focused elimination strategies. Only one is used for the analysis in this paper and briefly described in the result section in this paper.

The technical basis for ANACONDA, was developed at the Global Burden of Disease Group at the University of Melbourne, while all aspects of software design and development were carried out by the Public Health Computing group at the Swiss Tropical and Public Health Institute, University of Basel in Switzerland. Financial support was provided by Bloomberg Philanthropies Data for Health Initiative.

More information about ANACONDA, its methods, concepts and resources can be found in the tool itself Available at the CRVS Knowledge Gateway ([www.crvsgateway.info/ANACONDA](http://www.crvsgateway.info/ANACONDA)) together with supporting materials.
