# Supplementary material for: Are cause of death data fit for purpose? evidence from 20 countries at different levels of socio-economic development
Source: PLoS One. 2020 Aug 24;15(8):e0237539. doi: 10.1371/journal.pone.0237539 (PMC7446871; doi:10.1371/journal.pone.0237539)
Supplement: S2 File — (DOCX) [file pone.0237539.s002.docx]

# S2 File

# The ICD-10 composition of each of the four levels of Garbage Codes is given in the table below:

| **Garbage Code Severity Level** | **ICD-10 Codes** |
| --- | --- |
| Very High (Level 1) | A40, A40.0 - A40.3, A40.8 - A40.9, A41, A41.0 - A41.6, A41.8 - A41.9, A48.0, A48.3, A49.0 - A49.1, A59, A59.0, A59.8 - A59.9, A71, A71.0 - A71.1, A71.9, A74.0, B07, B07.0, B07.8 - B07.9, B30, B30.0 - B30.3, B30.8 - B30.9, B35, B35.0 - B35.6, B35.8 - B35.9, B36, B36.0 - B36.3, B36.8 - B36.9, B85, B85.0 - B85.4, B87, B87.0 - B87.4, B87.8 - B87.9, B88, B88.0 - B88.3, B88.8 - B88.9, B94.0, D50, D50.0, D50.9, D62, D62.0, D62.9, D63, D63.0, D63.8, D64, D64.1 - D64.4, D64.8 - D64.9, D65, D65.0, D65.9, D68, D69.9, E15 - E16, E50, E50.0 - E50.9, E64.1, E85.3 - E85.9, E86, E86.0 - E86.9, E87, E87.0 - E87.6, E87.8 - E87.9, F06.2 - F06.4, F07.2, F09, F09.0, F09.9, F17, F17.0 - F17.9, F20, F20.0 - F20.6, F20.8 - F20.9, F21 - F22, F22.0, F22.8 - F22.9, F23, F23.0 - F23.3, F23.8 - F23.9, F25, F25.0 - F25.2, F25.8 - F25.9, F26 - F28, F28.0, F29, F29.0, F29.9, F30, F30.0 - F30.4, F30.8 - F30.9, F31, F31.0 - F31.9, F32, F32.0 - F32.5, F32.8 - F32.9, F33, F33.0 - F33.4, F33.8 - F33.9, F34, F34.0 - F34.1, F34.8 - F34.9, F35 - F38, F38.0 - F38.1, F38.8, F39, F40, F40.0 - F40.2, F40.8 - F40.9, F41, F41.0 - F41.3, F41.8 - F41.9, F42, F42.0 - F42.2, F42.8 - F42.9, F43, F43.0 - F43.2, F43.8 - F43.9, F44, F44.0 - F44.9, F45, F45.0 - F45.4, F45.8 - F45.9, F46 - F48, F48.0 - F48.2, F48.8 - F48.9, F49, F51, F51.0 - F51.5, F51.8 - F51.9, F52, F52.0 - F52.9, F53, F53.0 - F53.1, F53.8 - F53.9, F54 - F55, F55.0 - F55.4, F55.8 - F55.9, F56 - F59, F60, F60.0 - F60.9, F61 - F62, F62.0 - F62.1, F62.8 - F62.9, F63, F63.0 - F63.3, F63.8 - F63.9, F64, F64.0 - F64.2, F64.8 - F64.9, F65, F65.0 - F65.6, F65.8 - F65.9, F66, F66.0 - F66.2, F66.8 - F66.9, F67 - F68, F68.0 - F68.1, F68.8, F69, F69.0, F70, F70.0 - F70.1, F70.8 - F70.9, F71, F71.0 - F71.1, F71.8 - F71.9, F72, F72.0 - F72.1, F72.8 - F72.9, F73, F73.0 - F73.1, F73.8 - F73.9, F74 - F78, F78.0 - F78.1, F78.8 - F78.9, F79, F79.0 - F79.1, F79.8 - F79.9, F80, F80.0 - F80.4, F80.8 - F80.9, F81, F81.0 - F81.3, F81.8 - F81.9, F82, F82.0, F83 - F84, F84.0 - F84.5, F84.8 - F84.9, F85 - F89, F89.0, F90, F90.0 - F90.2, F90.8 - F90.9, F91, F91.0 - F91.3, F91.8 - F91.9, F92, F92.0, F92.8 - F92.9, F93, F93.0 - F93.3, F93.8 - F93.9, F94, F94.0 - F94.2, F94.8 - F94.9, F95, F95.0 - F95.2, F95.8 - F95.9, F96 - F98, F98.0 - F98.6, F98.8 - F98.9, F99, F99.0, G06, G06.0 - G06.2, G07, G07.0, G08, G08.0, G32, G32.0, G32.8, G43, G43.0 - G43.9, G44, G44.0 - G44.2, G44.4 - G44.5, G44.8, G47, G47.0 - G47.2, G47.4 - G47.6, G47.8 - G47.9, G50, G50.0 - G50.1, G50.8 - G50.9, G51, G51.0 - G51.4, G51.8 - G51.9, G52, G52.0 - G52.3, G52.7 - G52.9, G53, G53.0 - G53.3, G53.8, G54, G54.0 - G54.9, G55, G55.0 - G55.3, G55.8, G56, G56.0 - G56.4, G56.8 - G56.9, G57, G57.0 - G57.9, G58, G58.0, G58.7 - G58.9, G59, G59.0, G59.8, G60, G60.0 - G60.3, G60.8 - G60.9, G62, G62.0 - G62.2, G62.8 - G62.9, G63, G63.0 - G63.6, G63.8, G64, G64.0, G65, G65.0 - G65.2, G80, G80.0 - G80.4, G80.8 - G80.9, G81, G81.0 - G81.1, G81.9, G82, G82.0 - G82.5, G82.9, G83, G83.0 - G83.5, G83.8 - G83.9, G89, G89.0 - G89.4, G91, G91.0 - G91.2, G91.4, G91.8 - G91.9, G92, G92.5 - G92.6, G92.9, G93.1 - G93.2, G93.4 - G93.6, G99, G99.0 - G99.2, G99.8, H00, H00.0 - H00.1, H01, H01.0 - H01.1, H01.8 - H01.9, H02, H02.0 - H02.9, H03, H03.0 - H03.1, H03.8, H04, H04.0 - H04.6, H04.8 - H04.9, H05, H05.2 - H05.5, H05.8 - H05.9, H06, H06.0 - H06.3, H07 - H09, H10, H10.0 - H10.5, H10.8 - H10.9, H11, H11.0 - H11.4, H11.8 - H11.9, H12 - H13, H13.0 - H13.3, H13.8, H14 - H15, H15.0 - H15.1, H15.8 - H15.9, H16, H16.0 - H16.4, H16.8 - H16.9, H17, H17.0 - H17.1, H17.8 - H17.9, H18, H18.0 - H18.9, H19, H19.0 - H19.3, H19.8, H20, H20.0 - H20.2, H20.8 - H20.9, H21, H21.0 - H21.5, H21.8 - H21.9, H22, H22.0 - H22.1, H22.8, H23 - H25, H25.0 - H25.2, H25.8 - H25.9, H26, H26.0 - H26.4, H26.8 - H26.9, H27, H27.0 - H27.1, H27.8 - H27.9, H28, H28.0 - H28.2, H28.8, H29, H30, H30.0 - H30.2, H30.8 - H30.9, H31, H31.0 - H31.4, H31.8 - H31.9, H32, H32.0, H32.8, H33, H33.0 - H33.5, H33.8, H34, H34.0 - H34.2, H34.8 - H34.9, H35, H35.0 - H35.9, H36, H36.0, H36.8, H37 - H39, H40, H40.0 - H40.6, H40.8 - H40.9, H41 - H42, H42.0, H42.8, H43, H43.0 - H43.3, H43.8 - H43.9, H44, H44.0 - H44.9, H45, H45.0 - H45.1, H45.8, H46, H46.0 - H46.3, H46.8 - H46.9, H47, H47.0 - H47.7, H47.9, H48, H48.0 - H48.1, H48.8, H49, H49.0 - H49.4, H49.8 - H49.9, H50, H50.0 - H50.6, H50.8 - H50.9, H51, H51.0 - H51.2, H51.8 - H51.9, H52, H52.0 - H52.7, H53, H53.0 - H53.9, H54, H54.0 - H54.9, H55, H55.0, H55.8, H56 - H57, H57.0 - H57.1, H57.8 - H57.9, H58, H58.0, H58.8 - H58.9, H59, H59.0 - H59.4, H59.8, H60, H60.0 - H60.6, H60.8 - H60.9, H61, H61.0 - H61.3, H61.8 - H61.9, H62, H62.0 - H62.4, H62.8, H65, H65.0 - H65.4, H65.9, H66, H66.0 - H66.4, H66.9, H67, H67.0 - H67.3, H67.8 - H67.9, H68, H68.0 - H68.1, H69, H69.0, H69.8 - H69.9, H71, H71.0 - H71.3, H71.9, H72, H72.0 - H72.2, H72.8 - H72.9, H73, H73.0 - H73.2, H73.8 - H73.9, H74, H74.0 - H74.4, H74.8 - H74.9, H75, H75.0, H75.8, H76 - H79, H80, H80.0 - H80.2, H80.8 - H80.9, H81, H81.0 - H81.4, H81.8 - H81.9, H82, H82.1 - H82.3, H82.9, H83, H83.0 - H83.3, H83.8 - H83.9, H84 - H87, H87.6, H88 - H89, H90, H90.0 - H90.8, H91, H91.0 - H91.3, H91.8 - H91.9, H92, H92.0 - H92.2, H93, H93.0 - H93.3, H93.8 - H93.9, H94, H94.0, H94.8, H95, H95.0 - H95.4, H95.8 - H95.9, H96 - H99, I26, I26.0, I26.9, I31.2 - I31.4, I46, I46.0 - I46.2, I46.6, I46.8 - I46.9, I50, I50.0 - I50.4, I50.8 - I50.9, I51.7, I67.4, I76, I95, I95.0 - I95.1, I95.8 - I95.9, J69, J69.0 - J69.1, J69.8 - J69.9, J80, J80.0, J80.9, J85, J85.0 - J85.3, J86, J86.0, J86.9, J93, J93.0 - J93.1, J93.8 - J93.9, J94.2, J96, J96.0 - J96.2, J96.4 - J96.5, J96.8 - J96.9, J98.1 - J98.3, K00, K00.0 - K00.9, K01, K01.0 - K01.1, K02, K02.0 - K02.9, K03, K03.0 - K03.9, K04, K04.0 - K04.9, K05, K05.0 - K05.6, K06, K06.0 - K06.2, K06.8 - K06.9, K07, K07.0 - K07.6, K07.8 - K07.9, K08, K08.0 - K08.5, K08.8 - K08.9, K09, K09.0 - K09.2, K09.8 - K09.9, K10, K10.0 - K10.3, K10.8 - K10.9, K11, K11.0 - K11.9, K12, K12.0 - K12.3, K13, K13.0 - K13.7, K14, K14.0 - K14.6, K14.8 - K14.9, K15.9, K16 - K19, K30, K65, K65.0 - K65.4, K65.8 - K65.9, K66, K66.0 - K66.1, K66.9, K71, K71.0 - K71.6, K71.8 - K71.9, K72, K72.0 - K72.1, K72.9, K75.0, L20, L20.0, L20.8 - L20.9, L21, L21.0 - L21.1, L21.8 - L21.9, L22 - L23, L23.0 - L23.9, L24, L24.0 - L24.9, L25, L25.0 - L25.5, L25.8 - L25.9, L26, L26.9, L27, L27.0 - L27.2, L27.8 - L27.9, L28, L28.0 - L28.2, L29, L29.0 - L29.3, L29.8 - L29.9, L30, L30.0 - L30.5, L30.8 - L30.9, L40, L40.0 - L40.5, L40.8 - L40.9, L41, L41.0 - L41.5, L41.8 - L41.9, L42 - L43, L43.0 - L43.3, L43.8 - L43.9, L44, L44.0 - L44.4, L44.8 - L44.9, L45 - L49, L49.0 - L49.9, L50, L50.0 - L50.6, L50.8 - L50.9, L52 - L53, L53.0 - L53.3, L53.8 - L53.9, L54, L54.0, L56, L56.0 - L56.2, L56.4 - L56.5, L57, L57.0 - L57.5, L57.8 - L57.9, L59, L59.0, L59.8 - L59.9, L60, L60.0 - L60.5, L60.8 - L60.9, L61 - L62, L62.0, L62.8, L63, L63.0 - L63.2, L63.8 - L63.9, L64, L64.0, L64.8 - L64.9, L65, L65.0 - L65.2, L65.8 - L65.9, L66, L66.0 - L66.4, L66.8 - L66.9, L67, L67.0 - L67.1, L67.8 - L67.9, L68, L68.0 - L68.3, L68.8 - L68.9, L70, L70.0 - L70.5, L70.8 - L70.9, L71, L71.0 - L71.1, L71.8 - L71.9, L72, L72.0 - L72.3, L72.8 - L72.9, L73, L73.0 - L73.2, L73.8 - L73.9, L74, L74.0 - L74.5, L74.8 - L74.9, L75, L75.0 - L75.2, L75.8 - L75.9, L76, L76.0 - L76.2, L76.8, L80 - L81, L81.0 - L81.9, L82, L82.0 - L82.1, L83 - L85, L85.0 - L85.3, L85.8 - L85.9, L86 - L87, L87.0 - L87.2, L87.8 - L87.9, L90, L90.0 - L90.6, L90.8 - L90.9, L91, L91.0, L91.8 - L91.9, L92, L92.0 - L92.3, L92.8 - L92.9, L94, L94.0 - L94.6, L94.8 - L94.9, L95, L95.0 - L95.1, L95.8 - L95.9, L96, L98.5 - L98.6, L98.8 - L98.9, L99, L99.0, L99.8, M04, M10, M10.0 - M10.4, M10.9, M11, M11.0 - M11.2, M11.8 - M11.9, M12, M12.0, M12.2 - M12.5, M12.8 - M12.9, M13, M13.0 - M13.1, M13.8 - M13.9, M14, M14.0 - M14.6, M14.8, M15, M15.0 - M15.4, M15.8 - M15.9, M16, M16.0 - M16.7, M16.9, M17, M17.0 - M17.5, M17.9, M18, M18.0 - M18.5, M18.9, M19, M19.0 - M19.2, M19.8 - M19.9, M20, M20.0 - M20.6, M21, M21.0 - M21.9, M22, M22.0 - M22.4, M22.8 - M22.9, M23, M23.0 - M23.6, M23.8 - M23.9, M24, M24.0 - M24.9, M25, M25.0 - M25.9, M26, M26.0 - M26.9, M27, M27.0 - M27.6, M27.8 - M27.9, M28 - M29, M37 - M39, M43.2 - M43.6, M43.8 - M43.9, M44 - M45, M45.0 - M45.9, M46, M46.0 - M46.5, M46.8 - M46.9, M47, M47.0 - M47.2, M47.8 - M47.9, M48, M48.0 - M48.5, M48.8 - M48.9, M49, M49.2 - M49.5, M49.8, M50, M50.0 - M50.3, M50.8 - M50.9, M51, M51.0 - M51.4, M51.8 - M51.9, M52 - M53, M53.0 - M53.3, M53.8 - M53.9, M54, M54.0 - M54.6, M54.8 - M54.9, M55 - M59, M60, M60.0 - M60.2, M60.8 - M60.9, M61, M61.0 - M61.5, M61.9, M62, M62.0 - M62.6, M62.8 - M62.9, M63, M63.0 - M63.3, M63.8, M64, M65.1 - M65.4, M65.8 - M65.9, M66, M66.0 - M66.5, M66.8 - M66.9, M67, M67.0 - M67.5, M67.8 - M67.9, M68, M68.0, M68.8, M69, M70, M70.0 - M70.9, M71, M71.2 - M71.5, M71.8 - M71.9, M72, M72.0 - M72.4, M72.8 - M72.9, M73, M73.8, M74 - M75, M75.0 - M75.5, M75.8 - M75.9, M76, M76.0 - M76.9, M77, M77.0 - M77.5, M77.8 - M77.9, M78 - M79, M79.0 - M79.9, M83, M83.0 - M83.5, M83.8 - M83.9, M84, M84.0 - M84.6, M84.8 - M84.9, M85, M85.0 - M85.6, M85.8 - M85.9, M86, M86.0 - M86.2, M86.5 - M86.9, M87.2 - M87.3, M87.8 - M87.9, M89.1 - M89.4, M90, M90.0 - M90.8, M91, M91.0 - M91.4, M91.8 - M91.9, M92, M92.0 - M92.9, M93, M93.0 - M93.2, M93.8 - M93.9, M94, M94.0 - M94.3, M94.8 - M94.9, M95, M95.0 - M95.5, M95.8 - M95.9, M96, M96.0 - M96.6, M96.8 - M96.9, M97 - M99, M99.0 - M99.9, N17, N17.0 - N17.2, N17.8 - N17.9, N19, N19.0, N19.9, N32.1 - N32.2, N32.8 - N32.9, N33, N33.0, N33.8, N35, N35.0 - N35.1, N35.8 - N35.9, N37, N37.0, N37.8, N39.3 - N39.4, N39.8, N42, N42.0 - N42.3, N42.8 - N42.9, N43, N43.0 - N43.4, N44.1 - N44.2, N44.8, N46, N46.0 - N46.1, N46.8 - N46.9, N47, N47.0 - N47.8, N48, N48.0 - N48.6, N48.8 - N48.9, N50, N50.0 - N50.1, N50.3, N50.8 - N50.9, N51, N51.0 - N51.2, N51.8, N52, N52.0 - N52.3, N52.8 - N52.9, N53, N53.1, N53.8 - N53.9, N61, N61.0, N61.9, N62 - N63, N63.0, N64, N64.0 - N64.5, N64.8 - N64.9, N82, N82.0 - N82.5, N82.8 - N82.9, N91, N91.0 - N91.5, N95, N95.1 - N95.3, N95.8 - N95.9, N97, N97.0 - N97.4, N97.8 - N97.9, R02, R02.0, R02.9, R03.1, R07.0, R08 - R09, R09.3, R11, R11.0 - R11.2, R11.9, R12, R12.0, R14, R14.0 - R14.3, R15, R15.0 - R15.2, R15.9, R19, R19.0 - R19.6, R19.8, R20, R20.0 - R20.3, R20.8 - R20.9, R21, R21.0, R22, R22.0 - R22.4, R22.7, R22.9, R23, R23.1 - R23.4, R23.8 - R23.9, R24, R24.0, R25, R25.0 - R25.4, R25.8 - R25.9, R26, R26.0 - R26.3, R26.8 - R26.9, R27, R27.0, R27.8 - R27.9, R28 - R29, R29.0 - R29.6, R29.8 - R29.9, R30, R30.0 - R30.1, R30.9, R32 - R33, R33.0, R33.8 - R33.9, R34, R34.0, R34.9, R35, R35.0 - R35.1, R35.8, R36, R36.0 - R36.1, R36.9, R37 - R39, R39.0 - R39.2, R39.8 - R39.9, R40, R40.0 - R40.4, R41, R41.0 - R41.4, R41.8 - R41.9, R42, R42.0, R43, R43.0 - R43.2, R43.8 - R43.9, R44, R44.0 - R44.3, R44.8 - R44.9, R45, R45.0 - R45.8, R46, R46.0 - R46.8, R47, R47.0 - R47.1, R47.8 - R47.9, R48, R48.0 - R48.3, R48.8 - R48.9, R49, R49.0 - R49.2, R49.8 - R49.9, R50, R50.0 - R50.1, R50.8 - R50.9, R51, R51.0, R52, R52.0 - R52.2, R52.9, R53, R53.0 - R53.2, R53.8, R54, R54.0, R54.9, R55, R55.0, R56, R56.0 - R56.1, R56.6, R56.8 - R56.9, R57, R57.0 - R57.1, R57.4, R57.6, R57.8 - R57.9, R58.0, R58.8 - R58.9, R59, R59.0 - R59.1, R59.9, R60, R60.0 - R60.1, R60.9, R61, R61.0 - R61.1, R61.9, R62, R62.0, R62.5, R62.7 - R62.9, R63, R63.0 - R63.6, R63.8, R64, R64.0, R64.9, R65, R65.1 - R65.2, R66 - R68, R68.0 - R68.3, R68.8, R69, R69.0, R69.9, R70, R70.0 - R70.1, R71, R71.0, R71.8, R72, R72.0, R72.9, R74, R74.0, R74.6, R74.8 - R74.9, R75, R75.0, R75.9, R76, R76.0 - R76.2, R76.8 - R76.9, R77, R77.0 - R77.2, R77.8 - R77.9, R78, R78.6 - R78.9, R79, R79.0 - R79.1, R79.8 - R79.9, R80, R80.0 - R80.3, R80.8 - R80.9, R81, R81.0, R82, R82.0 - R82.9, R83, R83.0 - R83.9, R84, R84.0 - R84.9, R85, R85.0 - R85.9, R86, R86.0 - R86.9, R87, R87.0 - R87.9, R88, R88.0, R88.8, R89, R89.0 - R89.9, R90, R90.0, R90.8, R91, R91.0 - R91.1, R91.8, R92, R92.0 - R92.2, R92.8, R93, R93.0 - R93.9, R94, R94.0 - R94.8, R96, R96.0 - R96.3, R97, R97.0 - R97.2, R97.8, R98, R98.0, R98.9, R99, R99.0, R99.9, U05, U07 - U09, U10 - U19, U20 - U29, U30 - U39, U40 - U49, U50 - U59, U60 - U69, U70 - U79, U80 - U81, U90 - U99, X40, X40.0 - X40.2, X40.4 - X40.9, X41, X41.0 - X41.9, X42, X42.0 - X42.9, X43, X43.0 - X43.9, X44, X44.0 - X44.9, X49, X49.0 - X49.9, X55, Y10, Y10.0 - Y10.9, Y11, Y11.0 - Y11.9, Y12, Y12.0 - Y12.9, Y13, Y13.0 - Y13.9, Y14, Y14.0 - Y14.9, Y16, Y16.0 - Y16.9, Y17, Y17.0 - Y17.9, Y18, Y18.0 - Y18.9, Y19, Y19.0 - Y19.9, Z00, Z00.0 - Z00.8, Z01, Z01.0 - Z01.9, Z02, Z02.0 - Z02.9, Z03, Z03.0 - Z03.9, Z04, Z04.0 - Z04.9, Z05 - Z08, Z08.0 - Z08.2, Z08.7 - Z08.9, Z09, Z09.0 - Z09.4, Z09.7 - Z09.9, Z10, Z10.0 - Z10.3, Z10.8, Z11, Z11.0 - Z11.6, Z11.8 - Z11.9, Z12, Z12.0 - Z12.9, Z13, Z13.0 - Z13.9, Z14, Z14.0 - Z14.1, Z14.8, Z15, Z15.0, Z15.8, Z17, Z17.0 - Z17.1, Z18, Z18.0 - Z18.3, Z18.8 - Z18.9, Z19, Z20, Z20.0 - Z20.9, Z21, Z21.0, Z22, Z22.0 - Z22.6, Z22.8 - Z22.9, Z23, Z23.0 - Z23.8, Z24, Z24.0 - Z24.6, Z25, Z25.0 - Z25.1, Z25.8, Z26, Z26.0, Z26.8 - Z26.9, Z27, Z27.0 - Z27.4, Z27.8 - Z27.9, Z28, Z28.0 - Z28.3, Z28.8 - Z28.9, Z29, Z29.0 - Z29.2, Z29.8 - Z29.9, Z30, Z30.0 - Z30.5, Z30.8 - Z30.9, Z31, Z31.0 - Z31.6, Z31.8 - Z31.9, Z32, Z32.0 - Z32.3, Z33, Z33.1 - Z33.2, Z34, Z34.0, Z34.8 - Z34.9, Z35, Z35.0 - Z35.9, Z36, Z36.0 - Z36.5, Z36.8 - Z36.9, Z37, Z37.0 - Z37.7, Z37.9, Z38, Z38.0 - Z38.8, Z39, Z39.0 - Z39.2, Z40, Z40.0, Z40.8 - Z40.9, Z41, Z41.0 - Z41.3, Z41.8 - Z41.9, Z42, Z42.0 - Z42.4, Z42.8 - Z42.9, Z43, Z43.0 - Z43.9, Z44, Z44.0 - Z44.3, Z44.8 - Z44.9, Z45, Z45.0 - Z45.4, Z45.8 - Z45.9, Z46, Z46.0 - Z46.9, Z47, Z47.0 - Z47.3, Z47.8 - Z47.9, Z48, Z48.0 - Z48.3, Z48.8 - Z48.9, Z49, Z49.0 - Z49.3, Z50, Z50.0 - Z50.9, Z51, Z51.0 - Z51.6, Z51.8 - Z51.9, Z52, Z52.0 - Z52.9, Z53, Z53.0 - Z53.2, Z53.8 - Z53.9, Z54, Z54.0 - Z54.4, Z54.7 - Z54.9, Z55, Z55.0 - Z55.4, Z55.8 - Z55.9, Z56, Z56.0 - Z56.9, Z57, Z57.0 - Z57.9, Z58, Z58.0 - Z58.9, Z59, Z59.0 - Z59.9, Z60, Z60.0 - Z60.5, Z60.8 - Z60.9, Z61, Z61.0 - Z61.9, Z62, Z62.0 - Z62.6, Z62.8 - Z62.9, Z63, Z63.0 - Z63.9, Z64, Z64.0 - Z64.4, Z65, Z65.0 - Z65.5, Z65.8 - Z65.9, Z66 - Z67, Z67.1 - Z67.4, Z67.9, Z68, Z68.1 - Z68.5, Z69, Z69.0 - Z69.1, Z69.8, Z70, Z70.0 - Z70.3, Z70.8 - Z70.9, Z71, Z71.0 - Z71.9, Z72, Z72.0 - Z72.6, Z72.8 - Z72.9, Z73, Z73.0 - Z73.6, Z73.8 - Z73.9, Z74, Z74.0 - Z74.3, Z74.8 - Z74.9, Z75, Z75.0 - Z75.5, Z75.8 - Z75.9, Z76, Z76.0 - Z76.5, Z76.8 - Z76.9, Z77, Z77.0 - Z77.2, Z77.9, Z78, Z78.0 - Z78.1, Z78.9, Z79, Z79.0 - Z79.5, Z79.8, Z80, Z80.0 - Z80.9, Z81, Z81.0 - Z81.4, Z81.8, Z82, Z82.0 - Z82.8, Z83, Z83.0 - Z83.7, Z84, Z84.0 - Z84.3, Z84.8, Z85, Z85.0 - Z85.9, Z86, Z86.0 - Z86.7, Z87, Z87.0 - Z87.8, Z88, Z88.0 - Z88.9, Z89, Z89.0 - Z89.9, Z90, Z90.0 - Z90.8, Z91, Z91.0 - Z91.6, Z91.8, Z92, Z92.0 - Z92.6, Z92.8 - Z92.9, Z93, Z93.0 - Z93.6, Z93.8 - Z93.9, Z94, Z94.0 - Z94.9, Z95, Z95.0 - Z95.5, Z95.8 - Z95.9, Z96, Z96.0 - Z96.9, Z97, Z97.0 - Z97.5, Z97.8, Z98, Z98.0 - Z98.6, Z98.8, Z99, Z99.0 - Z99.3, Z99.8 - Z99.9, ZA1 - ZA8, ZB0 |
| High (Level 2) | A14.9, A29, A30, A30.0 - A30.5, A30.8 - A30.9, A45, A45.9, A47 - A48, A48.8, A49, A49.3, A49.8 - A49.9, A61 - A62, A72 - A73, A76, A97, B08, B08.0 - B08.8, B09, B11 - B14, B28 - B29, B31, B31.9, B32, B32.3 - B32.4, B34, B34.0 - B34.4, B34.8 - B34.9, B61 - B62, B68, B68.0 - B68.1, B68.9, B73, B73.0 - B73.1, B74, B74.0 - B74.2, B76, B76.0 - B76.1, B76.8 - B76.9, B78, B78.0 - B78.1, B78.7, B78.9, B79, B80 - B81, B81.0 - B81.4, B81.8, B84, B92 - B94, B94.8 - B94.9, B95.6 - B95.8, B96, B96.0 - B96.8, B97, B97.0 - B97.3, B97.7 - B97.8, B98 - B99, B99.0, B99.8 - B99.9, D59, D59.4, D59.8 - D59.9, G44.3, G91.3, G93.0, G93.3, I10, I10.0, I10.9, I15, I15.0 - I15.2, I15.8 - I15.9, I27, I27.0, I27.2, I27.8 - I27.9, I28.9, I70, I70.0 - I70.1, I70.9, I74, I74.0 - I74.5, I74.8 - I74.9, I75, I75.0, I75.8, J81, J81.0 - J81.1, J90, J90.0, J94, J94.0 - J94.1, J94.8 - J94.9, K92.0 - K92.2, N70, N70.0 - N70.1, N70.9, N71, N71.0 - N71.1, N71.9, N73, N73.0 - N73.6, N73.8 - N73.9, N74, N74.0, N74.2 - N74.4, N74.8, R03, R03.0, R04, R04.0 - R04.2, R04.8 - R04.9, R05, R05.0, R06, R06.0 - R06.9, R09.0 - R09.2, R09.8, R10, R10.0 - R10.4, R10.8 - R10.9, R13, R13.0 - R13.1, R13.9, R16, R16.0 - R16.2, R17, R17.0, R17.9, R18, R18.0, R18.8 - R18.9, R23.0, R58, S00, S00.0 - S00.5, S00.7 - S00.9, S01, S01.0 - S01.5, S01.7 - S01.9, S02, S02.0 - S02.9, S03, S03.0 - S03.5, S03.8 - S03.9, S04, S04.0 - S04.9, S05, S05.0 - S05.9, S06, S06.0 - S06.9, S07, S07.0 - S07.1, S07.8 - S07.9, S08, S08.0 - S08.1, S08.8 - S08.9, S09, S09.0 - S09.3, S09.7 - S09.9, S10, S10.0 - S10.1, S10.7 - S10.9, S11, S11.0 - S11.2, S11.7 - S11.9, S12, S12.0 - S12.9, S13, S13.0 - S13.6, S13.8 - S13.9, S14, S14.0 - S14.6, S14.8 - S14.9, S15, S15.0 - S15.3, S15.7 - S15.9, S16, S16.1 - S16.2, S16.8 - S16.9, S17, S17.0, S17.8 - S17.9, S18 - S19, S19.7 - S19.9, S20, S20.0 - S20.4, S20.7 - S20.9, S21, S21.0 - S21.4, S21.7 - S21.9, S22, S22.0 - S22.5, S22.8 - S22.9, S23, S23.0 - S23.5, S23.8 - S23.9, S24, S24.0 - S24.6, S24.8 - S24.9, S25, S25.0 - S25.5, S25.7 - S25.9, S26, S26.0 - S26.1, S26.8 - S26.9, S27, S27.0 - S27.9, S28, S28.0 - S28.2, S29, S29.0, S29.7 - S29.9, S30, S30.0 - S30.3, S30.7 - S30.9, S31, S31.0 - S31.5, S31.7 - S31.8, S32, S32.0 - S32.5, S32.7 - S32.9, S33, S33.0 - S33.9, S34, S34.0 - S34.6, S34.8 - S34.9, S35, S35.0 - S35.5, S35.7 - S35.9, S36, S36.0 - S36.9, S37, S37.0 - S37.9, S38, S38.0 - S38.3, S39, S39.0, S39.6 - S39.9, S40, S40.0, S40.2, S40.7 - S40.9, S41, S41.0 - S41.1, S41.7 - S41.8, S42, S42.0 - S42.4, S42.7 - S42.9, S43, S43.0 - S43.9, S44, S44.0 - S44.5, S44.7 - S44.9, S45, S45.0 - S45.3, S45.7 - S45.9, S46, S46.0 - S46.3, S46.7 - S46.9, S47, S47.1 - S47.2, S47.9, S48, S48.0 - S48.1, S48.9, S49, S49.0 - S49.1, S49.7 - S49.9, S50, S50.0 - S50.1, S50.3, S50.7 - S50.9, S51, S51.0, S51.7 - S51.9, S52, S52.0 - S52.9, S53, S53.0 - S53.4, S53.7, S54, S54.0 - S54.3, S54.7 - S54.9, S55, S55.0 - S55.2, S55.7 - S55.9, S56, S56.0 - S56.5, S56.7 - S56.9, S57, S57.0, S57.8 - S57.9, S58, S58.0 - S58.1, S58.9, S59, S59.0 - S59.2, S59.7 - S59.9, S60, S60.0 - S60.5, S60.7 - S60.9, S61, S61.0 - S61.5, S61.7 - S61.9, S62, S62.0 - S62.9, S63, S63.0 - S63.7, S63.9, S64, S64.0 - S64.4, S64.7 - S64.9, S65, S65.0 - S65.5, S65.7 - S65.9, S66, S66.0 - S66.9, S67, S67.0 - S67.4, S67.8 - S67.9, S68, S68.0 - S68.9, S69, S69.7 - S69.9, S70, S70.0 - S70.3, S70.7 - S70.9, S71, S71.0 - S71.1, S71.7 - S71.8, S72, S72.0 - S72.4, S72.7 - S72.9, S73, S73.0 - S73.1, S74, S74.0 - S74.2, S74.7 - S74.9, S75, S75.0 - S75.2, S75.7 - S75.9, S76, S76.0 - S76.4, S76.7 - S76.9, S77, S77.0 - S77.2, S78, S78.0 - S78.1, S78.9, S79, S79.0 - S79.1, S79.7 - S79.9, S80, S80.0 - S80.2, S80.7 - S80.9, S81, S81.0, S81.7 - S81.9, S82, S82.0 - S82.9, S83, S83.0 - S83.9, S84, S84.0 - S84.2, S84.7 - S84.9, S85, S85.0 - S85.5, S85.7 - S85.9, S86, S86.0 - S86.3, S86.7 - S86.9, S87, S87.0, S87.8, S88, S88.0 - S88.1, S88.9, S89, S89.0 - S89.3, S89.7 - S89.9, S90, S90.0 - S90.5, S90.7 - S90.9, S91, S91.0 - S91.3, S91.7, S92, S92.0 - S92.5, S92.7, S92.9, S93, S93.0 - S93.6, S94, S94.0 - S94.3, S94.7 - S94.9, S95, S95.0 - S95.2, S95.7 - S95.9, S96, S96.0 - S96.2, S96.7 - S96.9, S97, S97.0 - S97.1, S97.8, S98, S98.0 - S98.4, S98.9, S99, S99.7 - S99.9, SO6.9, T00, T00.0 - T00.3, T00.6, T00.8 - T00.9, T01, T01.0 - T01.3, T01.6, T01.8 - T01.9, T02, T02.0 - T02.9, T03, T03.0 - T03.4, T03.8 - T03.9, T04, T04.0 - T04.4, T04.7 - T04.9, T05, T05.0 - T05.6, T05.8 - T05.9, T06, T06.0 - T06.5, T06.8, T07, T07.0, T08, T08.0, T09, T09.0 - T09.6, T09.8 - T09.9, T10, T10.0, T11, T11.0 - T11.6, T11.8 - T11.9, T12, T12.0 - T12.1, T13, T13.0 - T13.6, T13.8 - T13.9, T14, T14.0 - T14.9, T15, T15.0 - T15.1, T15.8 - T15.9, T16, T16.1 - T16.2, T16.9, T17, T17.0 - T17.5, T17.8 - T17.9, T18, T18.0 - T18.5, T18.8 - T18.9, T19, T19.0 - T19.4, T19.8 - T19.9, T20, T20.0 - T20.7, T21, T21.0 - T21.7, T21.9, T22, T22.0 - T22.7, T23, T23.0 - T23.7, T24, T24.0 - T24.7, T25, T25.0 - T25.7, T26, T26.0 - T26.9, T27, T27.0 - T27.7, T28, T28.0 - T28.9, T29, T29.0 - T29.7, T30, T30.0 - T30.7, T31, T31.0 - T31.9, T32, T32.0 - T32.9, T33, T33.0 - T33.9, T34, T34.0 - T34.9, T35, T35.0 - T35.7, T36, T36.0 - T36.9, T37, T37.0 - T37.5, T37.8 - T37.9, T38, T38.0 - T38.9, T39, T39.0 - T39.4, T39.8 - T39.9, T40, T40.0 - T40.9, T41, T41.0 - T41.5, T42, T42.0 - T42.8, T43, T43.0 - T43.6, T43.8 - T43.9, T44, T44.0 - T44.9, T45, T45.0 - T45.9, T46, T46.0 - T46.9, T47, T47.0 - T47.9, T48, T48.0 - T48.7, T48.9, T49, T49.0 - T49.9, T50, T50.0 - T50.9, T51, T51.0 - T51.3, T51.8 - T51.9, T52, T52.0 - T52.4, T52.8 - T52.9, T53, T53.0 - T53.7, T53.9, T54, T54.0 - T54.3, T54.9, T55, T55.0 - T55.1, T56, T56.0 - T56.9, T57, T57.0 - T57.3, T57.8 - T57.9, T58, T58.0 - T58.2, T58.8 - T58.9, T59, T59.0 - T59.9, T60, T60.0 - T60.4, T60.8 - T60.9, T61, T61.0 - T61.2, T61.7 - T61.9, T62, T62.0 - T62.2, T62.8 - T62.9, T63, T63.0 - T63.9, T64, T64.0, T64.8, T65, T65.0 - T65.6, T65.8 - T65.9, T66 - T67, T67.0 - T67.9, T68, T68.0, T68.7, T69, T69.0 - T69.1, T69.8 - T69.9, T70, T70.0 - T70.4, T70.8 - T70.9, T71, T71.0 - T71.2, T71.9, T73, T73.0 - T73.3, T73.8 - T73.9, T74, T74.0 - T74.4, T74.8 - T74.9, T75, T75.0 - T75.4, T75.8, T76, T76.0 - T76.3, T76.9, T78, T78.0 - T78.4, T78.8 - T78.9, T79, T79.0 - T79.9, T80, T80.0 - T80.6, T80.8 - T80.9, T81, T81.0 - T81.9, T82, T82.0 - T82.9, T83, T83.0 - T83.9, T84, T84.0 - T84.9, T85, T85.0 - T85.9, T86, T86.0 - T86.5, T86.8 - T86.9, T87, T87.0 - T87.6, T87.8 - T87.9, T88, T88.0 - T88.9, T90, T90.0 - T90.5, T90.8 - T90.9, T91, T91.0 - T91.5, T91.8 - T91.9, T92, T92.0 - T92.6, T92.8 - T92.9, T93, T93.0 - T93.6, T93.8 - T93.9, T94, T94.0 - T94.1, T95, T95.0 - T95.4, T95.8 - T95.9, T96, T96.0, T97, T97.0, T98, T98.0 - T98.3, TO7, W47 - W48, W63, W71 - W72, W76, W76.0 - W76.9, W82, W95 - W98, X07, X56, X59, X59.0 - X59.9, Y20, Y20.0 - Y20.9, Y21, Y21.0 - Y21.9, Y22, Y22.0 - Y22.9, Y23, Y23.0 - Y23.9, Y24, Y24.0 - Y24.9, Y25, Y25.0 - Y25.2, Y25.4 - Y25.9, Y26, Y26.0 - Y26.9, Y27, Y27.0 - Y27.9, Y28, Y28.0 - Y28.9, Y29, Y29.0 - Y29.9, Y30, Y30.0 - Y30.9, Y31, Y31.0 - Y31.9, Y32, Y32.0 - Y32.9, Y33, Y33.0 - Y33.9, Y34, Y34.0 - Y34.9, Y86, Y86.0, Y86.2, Y86.8, Y87, Y87.2, Y89, Y89.9, Y90, Y90.0 - Y90.9, Y91, Y91.0 - Y91.3, Y91.9, Y92, Y92.0 - Y92.9, Y93, Y93.0 - Y93.9, Y94 - Y98, Y98.0, Y99, Y99.0 - Y99.2, Y99.8, Y99.9 |
| Medium (Level 3) | A01, A49.2, A64, A64.0, A99, A99.0, B55, B55.9, B89, C14, C14.0 - C14.3, C14.8 - C14.9, C26, C26.0 - C26.2, C26.8 - C26.9, C27 - C29, C35 - C36, C39, C39.0, C39.8 - C39.9, C42, C46, C46.0 - C46.9, C55, C55.0 - C55.1, C55.9, C57.9, C59, C6, C63.9, C68, C68.9, C75.9, C76, C76.0 - C76.5, C76.7 - C76.9, C77, C77.0 - C77.5, C77.8 - C77.9, C78, C78.0 - C78.8, C79, C79.0 - C79.9, C8, C80, C80.0 - C80.2, C80.9, C87, C97, C97.0, C97.9, C98 - C99, D0, D00, D00.0, D01, D01.4 - D01.5, D01.7, D01.9, D02, D02.4, D02.9, D07, D07.3, D07.6, D08 - D09, D09.1, D09.7, D09.9, D10, D10.9, D13, D13.9, D14, D14.4, D17, D17.0 - D17.7, D17.9, D18, D18.0 - D18.1, D19, D19.0 - D19.1, D19.7, D19.9, D20, D20.0 - D20.1, D20.9, D21, D21.0 - D21.6, D21.9, D28, D28.9, D29, D29.9, D30, D30.9, D36.0, D36.9, D37, D37.0, D37.6 - D37.9, D38, D38.6, D39, D39.0, D39.7, D39.9, D4, D40, D40.9, D41, D41.9, D44, D44.9, D48, D48.7, D48.9, D49, D49.0 - D49.1, D49.5, D49.7 - D49.9, D54, D75.9, D79, D85, D87 - D88, D90 - D99, E07.8 - E07.9, E08, E08.0 - E08.6, E08.8 - E08.9, E17 - E19, E34.9, E35, E35.0 - E35.1, E35.8, E37 - E39, E47 - E49, E62, E69, E87.7, E90, E90.0 - E90.9, E91, E91.0 - E91.9, E92, E92.0 - E92.9, E93, E93.0 - E93.9, E94, E94.0 - E94.9, E95, E95.0 - E95.9, E96, E96.0 - E96.3, E96.5 - E96.9, E97, E97.0 - E97.1, E97.3 - E97.9, E98, E98.0 - E98.9, E99, E99.0 - E99.1, E99.3, E99.5 - E99.9, F04, F04.0, F05, F05.0 - F05.1, F05.8 - F05.9, F06, F06.0 - F06.1, F06.5 - F06.9, F07, F07.0, F07.8 - F07.9, F08, F50, F50.8 - F50.9, G09, G09.0, G09.9, G15 - G19, G27 - G29, G33 - G34, G38 - G39, G42, G48 - G49, G66 - G69, G74 - G79, G84 - G88, G93, G93.8 - G93.9, G94, G94.0 - G94.2, G94.8, G96, G96.0 - G96.1, G96.8 - G96.9, G98, G98.0, G98.8 - G98.9, I00.0, I03 - I04, I14, I16, I16.9, I17 - I19, I29, I29.9, I44, I44.0 - I44.9, I45, I45.0 - I45.6, I45.8 - I45.9, I49, I49.0 - I49.5, I49.8 - I49.9, I51, I51.6, I51.8 - I51.9, I52, I52.0 - I52.1, I52.8, I53 - I59, I90, I91.9, I92 - I93, I93.9, I94, I96, I96.0, I96.9, I98.4, I98.8, I99, I99.0, I99.8 - I99.9, ID5.9, J02.9, J03.9, J04.3, J06, J06.9, J40, J40.0, J40.9, J47, J47.0 - J47.1, J47.9, J48 - J49, J49.9, J50 - J59, J71, J71.2, J72 - J79, J81.9, J83, J85.9, J87 - J89, J90.9, J93.6, J97 - J98, J98.0, J98.4 - J98.6, J98.8 - J98.9, J99, J99.0 - J99.1, J99.8, K31.9, K32 - K34, K39, K47 - K49, K53 - K54, K63, K63.0 - K63.4, K63.8 - K63.9, K69, K75, K78 - K79, K84, K87, K87.0 - K87.1, K88 - K89, K92, K92.9, K93, K96 - K99, L06 - L07, L09, L15 - L19, L31 - L39, L69, L77 - L79, N09, N13, N13.0 - N13.9, N24, N28.8 - N28.9, N38, N39.9, N40, N40.0 - N40.3, N40.9, N54 - N59, N66 - N69, N78 - N79, N84, N84.2 - N84.3, N84.8 - N84.9, N85, N85.0 - N85.9, N86, N88, N88.0 - N88.4, N88.8 - N88.9, N89, N89.0 - N89.9, N90, N90.0 - N90.9, N92, N92.0 - N92.6, N93, N93.0, N93.8 - N93.9, N94, N94.0 - N94.6, N94.8 - N94.9, N95.0, O08, O08.0 - O08.9, O17 - O18, O18.0, O19, O27, O37 - O38, O38.4, O39, O49, O50 - O59, O78 - O79, O93 - O95, O95.9, P06, P16 - P18, P30 - P34, P34.2, P40 - P49, P62 - P69, P73, P79, P82, P85 - P89, P96.9, P97 - P99, P99.9, Q08 - Q09, Q10, Q10.0 - Q10.3, Q19, Q29, Q36.0 - Q36.1, Q36.9, Q46 - Q49, Q88, Q89.9, Q94, Q99.9, R00, R00.0 - R00.2, R00.8 - R00.9, R01, R01.0 - R01.2, R07, R07.1 - R07.4, R07.8 - R07.9, R31, R31.0 - R31.2, R31.9, R31.9 |
| Low (Level 4) | B54, B54.0, B64, B82, B82.0, B82.9, B83.9, E12, E12.0 - E12.9, E13, E13.0 - E13.9, E14, E14.0 - E14.9, G00, G00.9, G01, G01.0, G02, G02.0 - G02.1, G02.8, G03.9, I37.9, I42, I42.0, I42.9, I51.5, I64, I64.0 - I64.1, I64.9, I67, I67.8 - I67.9, I68, I68.8, I69, I69.4, I69.8 - I69.9, J07 - J08, J15.9, J17, J17.0 - J17.3, J17.8, J18, J18.0 - J18.2, J18.7 - J18.9, J19, J19.6, J22, J22.0, J22.9, J23 - J29, J64, J64.0, J64.9, P23, P23.5 - P23.6, P23.8 - P23.9, P37.3 - P37.4, R73, R73.0, R73.9, V87, V87.0 - V87.1, V87.4 - V87.9, V88, V88.0 - V88.1, V88.4 - V88.9, V89, V89.0 - V89.4, V89.9, V99, V99.0, Y09, Y09.0 - Y09.9, Y85, Y85.0, Y85.9, Y85.9 |
