# Supplementary material for: Are cause of death data fit for purpose? evidence from 20 countries at different levels of socio-economic development
Source: PLoS One. 2020 Aug 24;15(8):e0237539. doi: 10.1371/journal.pone.0237539 (PMC7446871; doi:10.1371/journal.pone.0237539)
Supplement: S3 File — (DOCX) [file pone.0237539.s003.docx]

S3 File

**Population numbers and overall mortality characteristics for selected countries**

| **Country** | **Population (millions)** | **Population 65+ (%)** | **Life expectancy Males (years)** | **Life expectancy Females (years)** | **< 5 mortality (deaths per 100,000)** |
| --- | --- | --- | --- | --- | --- |
| **Finland** | 5.5 | 20.3 | 78.6 | 84.3 | 2.2 |
| **Canada** | 36.0 | 16.1 | 79.9 | 84.0 | 5.4 |
| **Australia** | 23.9 | 15.0 | 80.2 | 84.6 | 3.9 |
| **Japan** | 128.0 | 26.0 | 81.1 | 87.2 | 2.6 |
| **France** | 65.7 | 18.9 | 79.8 | 85.7 | 3.9 |
| **United Kingdom** | 65.4 | 18.1 | 79.2 | 82.7 | 4.4 |
| **Turkey** | 78.3 | 7.8 | 75.2 | 83.1 | 14.2 |
| **Argentina** | 44.3 | 10.9 | 73.6 | 79.9 | 11.0 |
| **Iran** | 79.4 | 5.0 | 75.5 | 79.4 | 14.4 |
| **Jordan** | 10.6 | 3.8 | 77.9 | 81.1 | 14.4 |
| **Thailand** | 70.6 | 10.6 | 74.3 | 82.0 | 8.7 |
| **South Africa** | 55.0 | 5.1 | 62.8 | 69.7 | 33.6 |
| **Tunisia** | 11.4 | 7.6 | 76.2 | 80.8 | 10.5 |
| **Brazil** | 206.0 | 8.0 | 72.0 | 79.1 | 18.4 |
| **Columbia** | 50.6 | 7.0 | 77.5 | 82.7 | 14.2 |
| **Uzbekistan** | 32.2 | 4.2 | 67.1 | 73.8 | 23.8 |
| **Kyrgyzstan** | 6.4 | 4.3 | 69.1 | 76.3 | 20.1 |
| **Egypt** | 93.8 | 5.1 | 68.0 | 74.4 | 19.2 |
| **Nicaragua** | 6.1 | 5.1 | 76.9 | 80.7 | 15.2 |
| **Tajikistan** | 8.5 | 3.3 | 67.7 | 73.3 | 46.9 |

| ***Sources:*** |
| --- |
| **Population** |
| UN Population Division 2015 data. World Population Prospect 2017 |
| <https://population.un.org/wpp/Download/Standard/Population/> |
|  |
| **Life expectancy and <5 years mortality rate** |
| GBD 2017 country profiles observed estimates |
| [www.healthdata.org](http://www.healthdata.org/) |
