# Supplementary material for: Are cause of death data fit for purpose? evidence from 20 countries at different levels of socio-economic development
Source: PLoS One. 2020 Aug 24;15(8):e0237539. doi: 10.1371/journal.pone.0237539 (PMC7446871; doi:10.1371/journal.pone.0237539)
Supplement: S4 File — (DOCX) [file pone.0237539.s004.docx]

S4 File

**Garbage codes as a percentage of all deaths in 8 age groups for select countries for males and females**

| **Males** | **Age groups (years)** | | |  |  |  |  |  |  |
| --- | --- | --- | --- | --- | --- | --- | --- | --- | --- |
|  | **<5** | **5-9** | **10-19** | **20-24** | **25-29** | **30-49** | **50-69** | **70+** | **Total** |
| Finland | 4.8 | 12.5 | 6.1 | 18.9 | 23.0 | 20.7 | 9.5 | 5.8 | 7.7 |
| Canada | 12.4 | 18.3 | 28.2 | 33.9 | 33.9 | 24.1 | 16.2 | 21.0 | 20.0 |
| Australia | 24.8 | 22.7 | 18.4 | 20.2 | 23.9 | 27.1 | 16.6 | 21.4 | 20.7 |
| Japan | 25.7 | 22.9 | 14.7 | 15.4 | 15.6 | 20.2 | 20.1 | 35.0 | 31.5 |
| France | 16.2 | 34.8 | 45.5 | 49.2 | 43.7 | 33.7 | 26.3 | 33.1 | 31.4 |
| UK | 12.3 | 22.9 | 33.1 | 37.5 | 38.6 | 32.0 | 16.5 | 21.3 | 20.9 |
| Turkey | 17.1 | 40.4 | 40.4 | 39.6 | 36.3 | 28.1 | 22.1 | 31.8 | 28.1 |
| Argentina | 17.7 | 38.0 | 35.9 | 33.6 | 36.8 | 38.1 | 41.7 | 55.2 | 47.2 |
| Iran | 17.6 | 35.3 | 35.9 | 35.9 | 37.0 | 37.3 | 32.7 | 37.1 | 34.6 |
| Jordan | 22.2 | 57.8 | 55.6 | 56.5 | 56.5 | 38.0 | 35.1 | 40.7 | 38.0 |
| Thailand | 21.2 | 34.8 | 49.1 | 50.4 | 47.5 | 43.6 | 42.0 | 59.8 | 49.6 |
| South Africa | 35.0 | 51.9 | 48.9 | 52.4 | 46.8 | 41.0 | 49.0 | 56.5 | 47.2 |
| Tunisia | 33.5 | 58.9 | 64.1 | 79.1 | 71.5 | 53.9 | 45.9 | 57.2 | 52.5 |
| Brazil | 16.9 | 33.4 | 19.1 | 18.6 | 20.9 | 28.2 | 35.0 | 44.4 | 35.9 |
| Colombia | 14.5 | 26.2 | 14.7 | 13.0 | 13.3 | 16.3 | 20.5 | 25.4 | 21.1 |
| Uzbekistan | 37.1 | 36.3 | 30.0 | 25.3 | 24.5 | 22.4 | 22.6 | 23.2 | 24.1 |
| Kyrgyzstan | 15.3 | 34.0 | 40.5 | 34.1 | 35.0 | 27.3 | 21.1 | 13.7 | 20.4 |
| Egypt | 55.3 | 48.8 | 49.4 | 53.7 | 56.1 | 61.0 | 63.6 | 71.0 | 64.2 |
| Nicaragua | 23.9 | 29.5 | 29.3 | 30.5 | 22.7 | 23.1 | 22.8 | 27.5 | 25.1 |
| Tajikistan | 17.8 | 50.8 | 49.3 | 50.4 | 41.3 | 43.7 | 50.5 | 61.1 | 50.4 |

| **Females** | **Age groups (years)** | | |  |  |  |  |  |  |
| --- | --- | --- | --- | --- | --- | --- | --- | --- | --- |
|  | **<5** | **5-9** | **10-19** | **20-24** | **25-29** | **30-49** | **50-69** | **70+** | **Total** |
| Finland | 3.6 | 25.0 | 11.1 | 25.0 | 19.5 | 14.7 | 7.6 | 6.1 | 6.5 |
| Canada | 12.9 | 15.5 | 22.3 | 32.4 | 30.1 | 21.8 | 15.5 | 24.1 | 22.4 |
| Australia | 25.3 | 21.1 | 18.1 | 16.3 | 22.4 | 23.4 | 16.1 | 26.0 | 24.5 |
| Japan | 23.5 | 28.6 | 16.5 | 19.6 | 19.4 | 16.8 | 15.8 | 43.5 | 40.3 |
| France | 15.3 | 24.3 | 38.1 | 47.1 | 38.2 | 26.7 | 23.0 | 39.1 | 36.6 |
| UK | 14.2 | 25.0 | 30.6 | 31.3 | 33.6 | 26.4 | 16.1 | 26.8 | 25.3 |
| Turkey | 18.5 | 39.6 | 36.5 | 30.0 | 30.8 | 24.7 | 29.0 | 39.3 | 35.7 |
| Argentina | 17.5 | 36.1 | 36.2 | 38.4 | 36.7 | 33.8 | 41.0 | 61.4 | 54.0 |
| Iran | 18.0 | 37.9 | 39.8 | 37.4 | 37.5 | 33.1 | 34.2 | 39.8 | 36.3 |
| Jordan | 22.7 | 52.2 | 42.4 | 35.5 | 36.0 | 29.8 | 39.4 | 44.5 | 38.5 |
| Thailand | 21.7 | 42.5 | 46.0 | 45.1 | 42.2 | 37.4 | 42.7 | 65.1 | 55.5 |
| South Africa | 36.6 | 39.7 | 41.8 | 44.2 | 31.4 | 33.3 | 53.0 | 77.6 | 50.8 |
| Tunisia | 34.0 | 52.6 | 66.3 | 57.1 | 62.9 | 46.8 | 50.0 | 64.6 | 57.6 |
| Brazil | 17.2 | 33.8 | 29.6 | 27.8 | 27.4 | 29.5 | 36.5 | 49.0 | 42.4 |
| Colombia | 15.5 | 26.4 | 21.5 | 18.5 | 17.0 | 18.8 | 21.4 | 28.6 | 25.1 |
| Uzbekistan | 38.8 | 42.6 | 29.1 | 24.9 | 21.9 | 20.4 | 21.9 | 24.4 | 24.4 |
| Kyrgyzstan | 15.2 | 44.8 | 26.9 | 23.9 | 30.9 | 21.2 | 16.7 | 13.9 | 16.0 |
| Egypt | 54.8 | 50.1 | 57.0 | 59.3 | 60.8 | 58.6 | 66.2 | 74.7 | 68.1 |
| Nicaragua | 21.8 | 43.1 | 30.0 | 24.4 | 15.6 | 21.7 | 22.8 | 30.3 | 26.3 |
| Tajikistan | 21.0 | 53.5 | 47.5 | 43.2 | 40.9 | 36.8 | 50.8 | 64.4 | 52.6 |
